# Female university students’ fertility intentions and their psychosocial factors

**DOI:** 10.1186/s12889-024-18121-9

**Published:** 2024-03-04

**Authors:** Penghao Qiao, Yiming Li, Yixuan Song, Xi Tian

**Affiliations:** College of Economics and Management, Nanjing Agricultural University, Xiaolingwei Street, Nanjing, 210095 Jiangsu China

**Keywords:** Female university students, Fertility intention, Psychosocial factors

## Abstract

**Background:**

Raising the birth rate can effectively increase the resulting labour supply and minimise the adverse impact of an ageing population on high-quality economic development since the demographic dividend is rapidly declining. The Chinese government has a “three-child” policy in place, yet the fertility rate is still falling. This study intends to investigate the present fertility intentions of female university students and assess the extent to which feminism has affected their intentions. It will next investigate the degree to which and the mechanisms by which the psychosocial factors have an impact on those intentions.

**Methods:**

A cross-sectional survey of female university students was conducted in Nanjing, China, from February to March 2023. To assure the representativeness of the sample, a technique of stratified proportional sampling, PPS sampling, and convenience sampling was utilized. A total of 1124 valid samples were acquired from female university students in 15 comprehensive universities. The data were mined and analysed by SPSS (version 24.0) and AMOS (version 24.0) software.

**Results:**

Overall female university students’ fertility intentions are low at this stage, with more than half (53.55%) of them having no clear desire to have children. The level of feminist identity significantly negatively affected the Intensity of desire to have children (-0.32) and child-number desires (-0.7). Psychosocial factors had a greater degree of influence on fertility intentions. The direct effect of the level of feminist identity and the perception of fertility hindrances on childbearing desires was -0.63 and -0.50 respectively, and the direct effect of the perception of fertility supports on childbearing intentions was 0.79.

**Conclusion:**

The level of feminist identity is significantly and negatively related to childbearing desires. Psychosocial factors have a greater degree of influence on fertility intentions, with the level of feminist identity, the perception of fertility hindrances and the perception of fertility supports all significantly impacting fertility intentions. The findings of this study emphasise the importance of the government providing a full range of social security and employers providing better employee benefits to promote a fertility-friendly society.

## Introduction

The population has a crucial role in fostering high-quality economic growth in addition to serving as the backbone of social development. Scholars and policymakers in China as well as other countries have focused heavily on low birth rates and population ageing. Since 2013, China’s overall fertility rate and percentage of people of working age have been quickly dropping. Despite the government’s implementation of the three-child policy in 2021, the fertility intentions of women of reproductive age have remained low [1–5]. Due to China’s low birth rate, the country’s labor supply will continue to fall and its population will age more rapidly. These factors have already been recognized as long-term contributors to the country’s slow economic development and lack of momentum [6–8].

When it comes to their intentions about fertility, university students, as a unique subset of youth, have a greater impact on the actual birth results. Previous studies of college students in Denmark, South Korea, and Saudi Arabia have revealed that although the majority of females are willing to become parents, most of them also intend to put off having children [9–11]. Studies on reproductive intentions in China have mostly examined married individuals [12–16], and the few sociological surveys on university students-the majority of whom are male and female [17–19]-do not accurately represent the fertility goals of any one gender.

Therefore, the purpose of this study is to investigate the fertility intentions of female university students in Nanjing, Jiangsu Province, and the related influencing factors, to discuss the gap between the current low fertility rate and the state’s strong encouragement of childbearing, and to provide practical suggestions to the government on how to create a friendly environment for childbearing.

### Theoretical models of fertility intentions

Economic Theory of Fertility situates the determinants of birth rates within the microeconomic theoretical structure of analysis. Harvey Leeibenstein was the first to suggest that when couples decide on the desired number of children they want to be born, this will equalise the positive and negative utilities associated with a finite number of children [20]; Gary S. Becker builds on Leeibenstein’s analysis to illustrate that, with a certain level of household income, it is necessary to have fewer children and raise them in order to purchase and enjoy a larger number of consumer goods [21]; R. A. Easterlin argued that the supply and demand for children generate positive and negative utility with modernisation [22], complementing the theories of Leeibenstein and Becker.

The Theory of Planned Behaviour in fertility intentions states that fertility intentions are not only rooted in rational trade-offs from an economic perspective, but are also governed by subjective emotions and beliefs [23].The TDIB model of fertility intentions proposed by Millier integrates motivations, desires and intentions into a system of indicators, which to some extent remedies some of the shortcomings of the Theory of Planned Behavior in Fertility Intentions [24]. He argues that the transition from fertility intentions to fertility behaviours follows the “motivations-desires-intentions-behaviors-outcomes” approach.With desires being the number of children one desires, including both the “child-number desires” and the “intensity of desire to have children”. The intensions is a decision made by individuals and families after weighing their fertility desires, the realities of their economic situation, work and health, etc. It has a strong relevance to reality.

### Measurement scales and factors influencing fertility intentions

Researchers typically utilise the “child-number desires” or the “child-number intentions under hypothetical conditions” to gauge people’s fertility intentions, according to the more reliable practise of fertility intention surveys in China.

Feng Xiaotian and Zheng Zhenzhen, on the other hand, contend that the “child-number desires” measures only people’s subjective “perceptions”, “understanding” or “notions” of fertility, and do not accurately reflect the actual number of children people choose to have in real life, taking into account specific personal and family conditions. At the same time, the “child-number intentions under hypothetical conditions” is based on the assumption that there is no policy in reality where there is a family planning policy, which is not a good reflection of the real intentions of people.But in comparison, the former is further away from people’s actual fertility intentions than the latter [25, 26].Regarding the factors affecting fertility intentions, there are factors at the societal level, including political, economic, cultural, policy and housing prices, as well as factors at the family and individual levels, including the family’s income, the education level of individuals of childbearing age, their energy, attitudes to and preferences for childbearing, and self-assessment of childbearing and parenting abilities and resources [27–40].

## Methods

### Design

A one-month cross-sectional survey of female university students was conducted in Nanjing, China, from 10 February to 10 March 2023, and all participants were voluntary participants.

### Sample and setting

The survey, which covered 51 campuses, was directed at female university students in Nanjing. These 51 universities were divided up into different teaching units, majors, and classes in order to provide a more representative sample. The survey was subsequently conducted on the female university students in the classes selected. First, 15 institutions were chosen using a stratified proportional sampling technique. Next, PPS sampling was used to choose teaching units, majors, and classes one at a time. Finally, a convenience sampling technique was used to disseminate the questionnaire using a combination of online and offline techniques. Based on the sample completed by the pre-survey questionnaire as the estimation object, the formula for the optimal sample size $n_0$ before correction was:

1 $$\begin{aligned} n_0 = \frac{t^2pQ(p)/d^2}{1+\frac{1}{N}\big [\frac{t^2pQ(p)}{d^2}-1\big ]} \end{aligned}$$

Where *N* is the overall number of university students in Nanjing, set the confidence level at 95%, according to the standard normal distribution table we can get $t=1.96$; *d* is the acceptable sampling limit error, take the value of 4%; *p* is the sample proportion, according to the pre-survey result $p=0.5$. We can approximate the optimal sample size as:

2 $$\begin{aligned} n_0 =\frac{t^2p\left( 1-p\right) }{d^2}{} & {} = \frac{1.96^2\times 0.5\times \left( 1-0.5\right) }{0.04^2} \nonumber \\{} & {} = 600.25 \end{aligned}$$

To retain integers, $n_0=601$ was taken, and due to the complexity of the sampling scheme, it was difficult to calculate the actual design effect deff. By combining the results of the pre-study with the literature, the design effect was determined to be 1.5, and the actual number of valid samples that should be recovered was approximately 902.

3 $$\begin{aligned} n = n_0 \times \text {deff} = 601 \times 1.5 \approx 902 \end{aligned}$$

Based on the above formula, we determined the final optimal sample size to be 902. Taking into account the invalidity of the sample due to invalid questionnaires or unsuitability as a sample, we estimated the actual number of questionnaires to be placed by using the invalid proportion of 10% from the pre-survey, which is $902 \div 0.9 \approx 1003$.

At the survey stage, in order to ensure the authenticity of the questionnaire filling, after fully explaining the content of the questionnaire and the precautions to the participants, the survey was carried out in the form of on-site distribution of questionnaires and supervision, and the time for the participants to fill in the questionnaire should not be less than 10 minutes. Secondly, after filling out the questionnaires, the participants should give the questionnaires to the on-site staff to recover the questionnaire data. Finally, the staff summarised all the completed questionnaires and found that a total of 1,500 questionnaires were distributed in this study and 1,308 were recovered, with a recovery rate of 87.20%. In addition, in order to improve the accuracy of the data, in the data pre-processing, through descriptive statistics, numerical conversion, missing value processing and other methods, 184 samples were excluded, such as the key information was not available, incomplete, randomly filled out, missing data, contradictory answers, etc., and 1,124 valid questionnaires were obtained (which was greater than the calculated required sample size of 1,003), with an effective rate of 85.93%.

### Questionnaires

The questionnaire consisted of 55 questions in four dimensions: sociodemographic characteristics, fertility intentions, perceptions of fertility hindrances and fertility supports, and level of Feminist Identity. Specifically, perceptions of fertility hindrances and fertility supports, along with the degree of feminist identity, were employed to assess the psychosocial status of participants.

### Sociodemographic characteristics

The data was collected using a sociodemographic questionnaire prepared by the research team. It collected information on age, university system, major, education level, domicile address and only child status.

### Fertility intentions

**Fig. 1:**
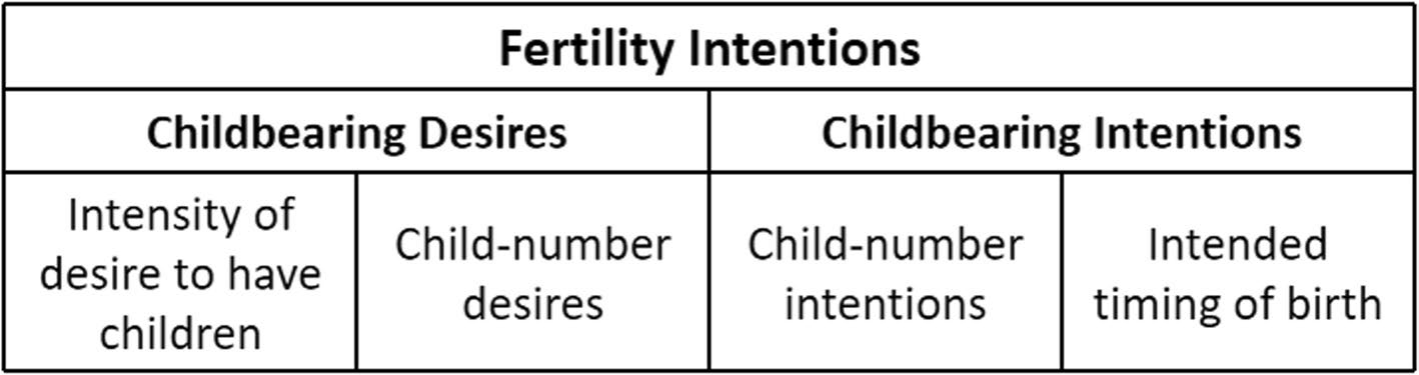
Fertility intention

The TDIB model of “motivations-desires-intentions-behaviors-outcomes” is relatively sound and scientific. As motivations is at the subconscious level, while childbearing desires and childbearing intentions are at the conscious level, the TDIB model uses “childbearing desires” and “childbearing intentions” to measure “fertility intentions” in this study, where “childbearing desires” includes “intensity of desire to have children” and “child-number desires”, which are measured by the questions “Whether you want to have children?” and “How many children do you think is the ideal number for an average family, regardless of fertility policies and other conditions?” In contrast, the “childbearing intentions” includes two indicators: “child-number intentions” and “intended timing of birth”, which are measured by the question “How many children do you plan to have?” and “If you are planning to have a baby, when will you choose to have it?” The theoretical framework of fertility intention is shown in Fig. 1.

### Perceptions of fertility hindrances and fertility supports

Perceptions of fertility hindrances include aspects such as financial stress, and perceptions of fertility supports include five aspects: financial supports, physical supports, timing supports, service provisions and employment protections. The Likert 5-point scoring was adopted, and the degree of agreement for each item from weak to strong was calculated from 1 to 5 points.

### Level of feminist identity

The level of feminist identity was measured using a modified FIDS scale(Feminist Identity Development Scale) based on the internationally recognized FIC scale (the Feminist Identity Composite) according to the Chinese reality, which contains 5 subscales, namely the Passive Acceptance Subscale (5 items), the Awareness Subscale (5 items), the Integration Development Subscale (4 items), the Integration Subscale (5 items) and the Active Engagement Subscale (8 items), the Likert 5-point scoring was adopted, and the degree of agreement for each item from weak to strong was calculated from 1 to 5 points. Which subscale had the highest average score indicated that the participant was at that stage in the development of that feminist identity development [41].

### Questionnaire testing

The overall reliability of the questionnaire in the pre-survey is 0.929, which is more than 0.70, indicating that the structure of the questionnaire and the design of the options are more scientific and reasonable, and the overall consistency of the questionnaire is very high. Since two scales were set up in this survey, namely, “Perceptions of fertility hindrances and fertility supports” and “Level of Feminist Identity”, in order to ensure the validity of the test results, the reliability test should be conducted separately. The Cronbach’s alpha of the two scales in the pre-survey was 0.952 and 0.907 respectively, and the reliability of each scale was greater than 0.70, indicating that the questionnaire had internal consistency; after the questionnaire was improved, the Cronbach’s alpha of the two scales in the formal survey was 0.951 and 0.847 respectively, which were more than 0.70, meaning that the design of the questionnaire was scientific and reasonable.Analysed by the results of KMO test and Bartlett’s Spherical Test, the KMO value of the questionnaire in the pre-survey was 0.875, which was more than 0.70, and it was very suitable for factor analysis. The results of confirmatory factor analysis(CFA) showed that $CMIN/DF=3.837$, $RMSEA=0.056$, $ITL=0.890$, $TLI=0.940$, $CFI=0.981$, indicating that the structural validity of the scale was good; after the questionnaire was improved, the KMO value of the questionnaire in the formal survey was 0.9345, which was more than 0.70, and it was very suitable for the factor analysis. The results of confirmatory factor analysis(CFA) showed that $CMIN/DF=1.656$, $RMSEA=0.044$, $ITL=0.960$, $TLI=0.950$, $CFI=0.960$, indicating excellent structural validity of the scale. The results showed that the research tool has good reliability and validity in this study.

### Data analyses

The questionnaire data were pre-processed by screening and assignment and then exported in numerical form to Excel, where the data were imported into SPSS 24.0 and AMOS 24.0 for statistical analysis. We used descriptive analyses to illustrate the sociodemographic characteristics, four indicators of fertility intentions, perceptions of fertility hindrances and fertility supports among female university students, respectively. For the measurement of the relationship between the level of feminist identity and fertility intentions, logistic models were developed, diagnosed by covariance, and then analysed by multinomial logistic regression and ordinal logistic regression. After analysing the influencing factors, a Structural Equation Model (SEM) was developed using AMOS to conduct a pathway analysis.

### Ethics statement

Ethical review and approval were performed by Ethics Committee of College of Economics and Management of Nanjing Agricultural University (Project Number NAU-CEM202301). A cover letter was presented to respondents to explain the aim and process of the study before the questionnaire was shared. After participants read the contents of the consent form and agreed to participate in the study, they checked “consent” button and began to fill in the questionnaire. Participation in this study was voluntary, anonymous and confidential. To maintain anonymity, participants were asked not to provide their name or telephone number. The research was performed in accordance with the Declaration of Helsinki. Informed consent was obtained from all participants during the interview. All data collected were treated anonymously and confidentially.

## Results

### Participants’ characteristics

There was a more balanced proportion of participants in terms of age, mostly between 20 and 23 years old, a balanced type of university to which they belonged, a good distribution of majors and grades, and a relatively equal distribution of the sample in terms of only child status and household status. The sociodemographic characteristics of the respondents are summarised in Table 1.

**Table 1:** Sociodemographic characteristics of participants ($N = 1124$)

Characteristics		Total N(%)
Age	Under 19 years old	367(32.65)
	20-23	547(48.67)
	24-26	151(13.43)
	27-29	18(1.60)
	Over 30 years old	41(3.65)
Type of school	“Double First-Class” university	397(35.32)
	General Universities	411(36.57)
	“Three-year college education”	316(28.11)
Major	Bachelor of Law	21(1.87)
	Bachelor of Engineering	165(14.68)
	Bachelor of Management	163(14.50)
	Bachelor of Education	45(4.01)
	Bachelor of Economics	106(9.43)
	Bachelor of Science	119(10.59)
	Bachelor of History	26(2.31)
	Bachelor of Agriculture	98(8.72)
	Bachelor of Literature	151(13.43)
	Bachelor of Medicine and Surgery	155(13.79)
	Bachelor of Fine Art	27(2.40)
	Bachelor of Philosophy	13(1.16)
	Others	35(3.11)
Grade	Freshman	161(14.32)
	Sophomore	297(26.42)
	Junior	331(29.45)
	Senior	145(12.90)
	First year of graduate school	42(3.74)
	Second year of graduate school	47(4.18)
	Third year of graduate school	27(2.41)
	First year of PhD program	46(4.09)
	Second year of PhD program	22(1.96)
	Third year of PhD program	6(0.53)
Place of birth	City district	520(46.26)
	Not in the city district	604(53.74)
Only child	Yes	555(49.38)
	No	569(50.62)

The group Under 19 years old in this paper has an age range of 16-19 years old

### Participants’ fertility intentions

Only 46.45% of the respondents had a desire to have children, not more than half of them. Excluding fertility policies and other conditions, participants considered 1.47 children to be the ideal number for the average family. However, when asked how many children they would like to have, the number of children they would like to have is only 1.03. The difference between the child-number intentions and the child-number desires reflects to some extent the perception of female university students of factors that hinder childbearing, possibly taking into account their financial, time, energy and other factors, the larger the difference, the greater the degree of influence of realistic factors on the fertility intentions. The female university students’ intended timing of birth is generally later in life, with nearly 80% choosing to have children after the age of 27. The fertility intention of total participants are summarised in Table 2.

**Table 2:** Sociodemographic characteristics of participants ($N = 1124$)

Variables		Total N(%)
Intensity of desire to have children	very undesired	214(19.03)
	undesired	187(16.63)
	uncertain	201(17.89)
	slightly desired	324(28.83)
	strongly desired	198(17.62)
Child-number desires	0	95(8.45)
	1	434(38.61)
	2	572(50.89)
	3	23(2.05)
Child-number intentions	0	324(28.83)
	1	458(40.75)
	2	328(29.18)
	3	14(1.24)
Intended timing of birth	20-23	28(2.50)
	24-26	218(19.40)
	27-29	541(48.13)
	Over 30 years old	337(29.92)

### Participants’ perceptions of fertility hindrances and fertility supports

**Fig. 2:**
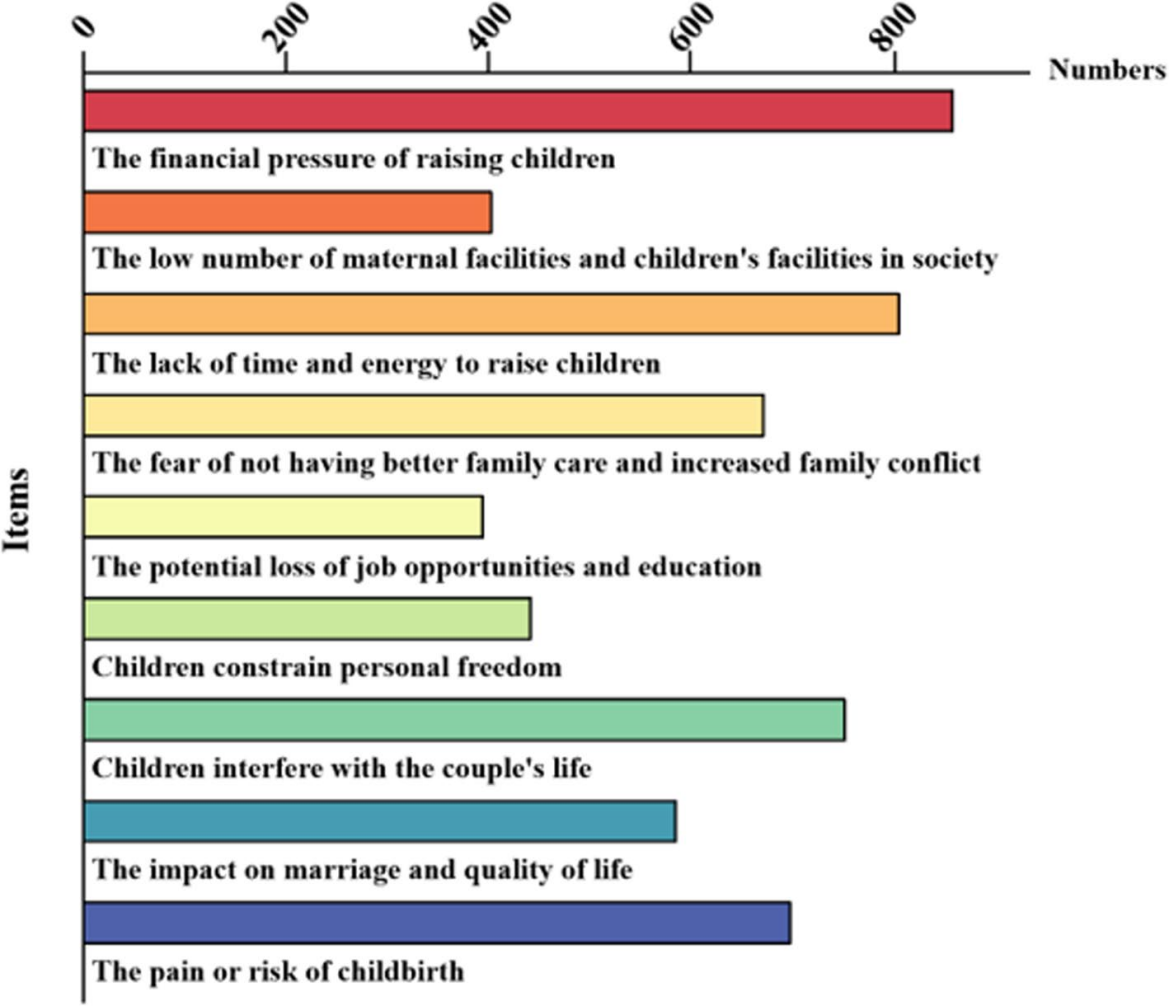
Perceptions of fertility hindrances

Among the hindrances to childbirth, financial pressure and lack of time and energy to raise children had the greatest impact on respondents, while the impact of the pain and risks of childbirth on female university students cannot be ignored. Female university students’ perceptions of fertility hindrances are shown in Fig. 2.

**Fig. 3:**
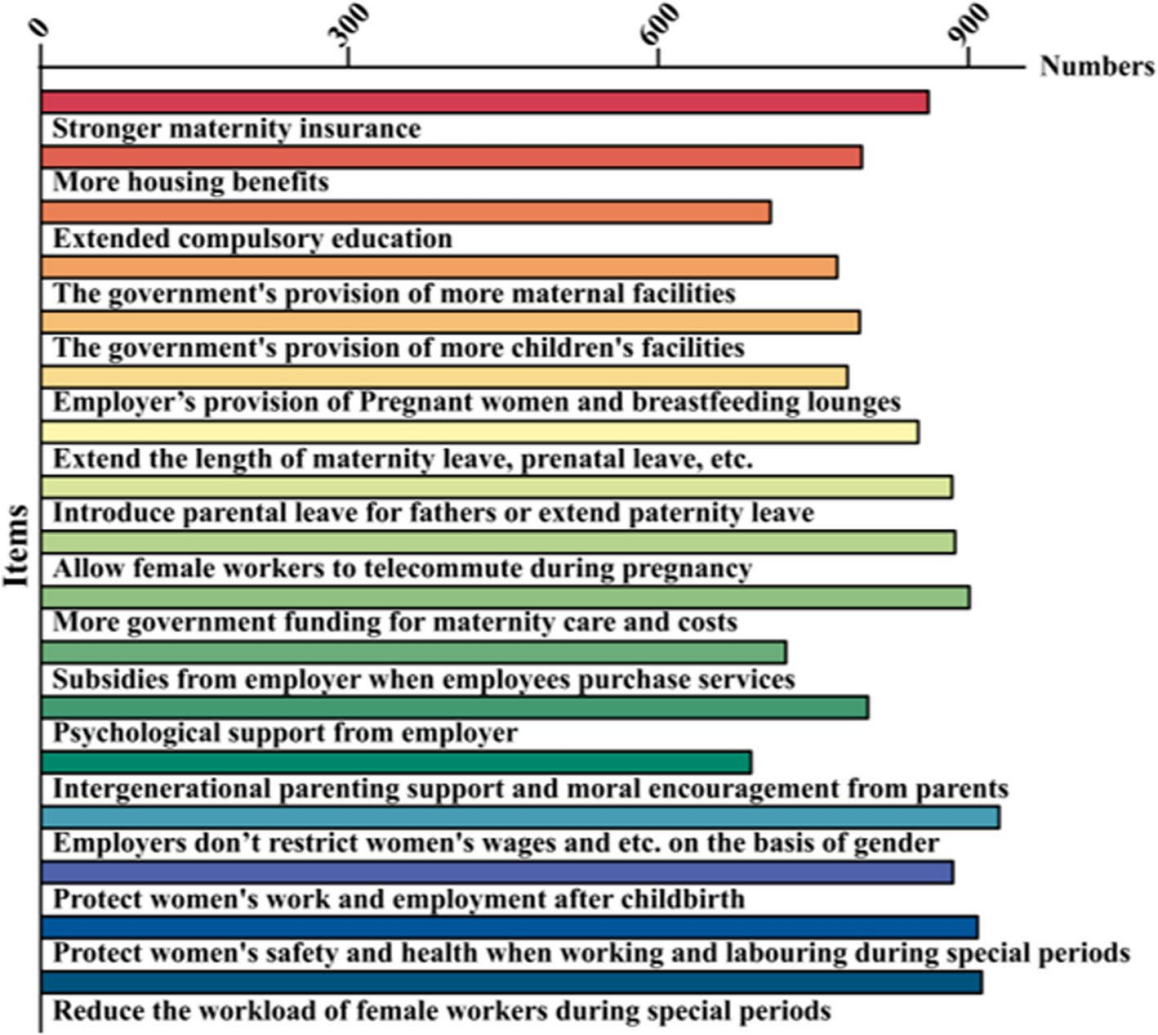
Perceptions of fertility supports

Among the external support measures for childbirth, employers don’t restrict women’s wages, salaries, career development and marital status on the basis of gender, protect women’s safety and health when working and labouring during special periods such as pregnancy and childbirth, protect women’s work and employment after childbirth and more government funding for maternity care and costs are the four most important support measures for female students in childbirth, all of which over 80% of them believe that they can increase their fertility intentions. Female university students’ perceptions of fertility supports are shown in Fig. 3.

### Statistical models

As the level of feminist identity is a sociopsychological factor and childbearing intentions is mainly influenced by the reality of the situation, it is necessary to use childbearing desires as the dependent variable when assessing the impact on fertility intentions, which contains two indicators, intensity of desire to have children and the child-number desires, and as both variables contain analogous relationships, each implying an intrinsic order of childbearing desires from weak to strong, logistic regression model was chosen for the regression analysis in this study. The general expressions of the model are as follows.

When the dependent variable is the intensity of desire to have children:

4 $$\begin{aligned} \text {Logit(P1)}= \;{} & {} \alpha +\beta _{\mathrm {1\ }}\textrm{X}_{\textrm{11}}+\beta _2 X_{12}+\beta _3 X_{13}+\beta _4 X_{14} \nonumber \\{} & {} +\beta _5 X_{21}+\beta _6 X_{22}+\beta _7 X_3+\beta _8 X_4 \nonumber \\{} & {} +\beta _9 X_5 \end{aligned}$$

When the dependent variable is the child-number desires:

5 $$\begin{aligned} \text {Logit(P2)}= \;{} & {} \alpha +\beta _{\mathrm {1\ }}\textrm{X}_{\textrm{11}}+\beta _2 X_{12}+\beta _3 X_{13}+\beta _4 X_{14} \nonumber \\{} & {} +\beta _5 X_{21}+\beta _6 X_{22}+\beta _7 X_3+\beta _8 X_4 \nonumber \\{} & {} +\beta _9 X_5 \end{aligned}$$

After a parallel line test, multinomial logistic regression was chosen when the dependent variable was intensity of desire to have children, and ordinal logistic regression was used when the dependent variable was child-number desires. The variable definitions are summarised in Table 3.

**Table 3:** Variable definition

Variables	Definition
Age	$$x_{11}=\left\{ \begin{array}{ll} 1&{} 20-23 \\ 0&{} \text {Others} \end{array}\right.$$
	$$x_{12}=\left\{ \begin{array}{ll} 1&{} 24-26 \\ 0&{} \text {Others} \end{array}\right.$$
	$$x_{13}=\left\{ \begin{array}{ll} 1&{} 27-29 \\ 0&{} \text {Others} \end{array}\right.$$
	$$x_{14}=\left\{ \begin{array}{ll} 1&{} \text {Over 30 years old} \\ 0&{} \text {Others} \end{array}\right.$$
	Group under 19 years old set as dummy variable basic type (Anchoring group)
Education	$$x_{21}=\left\{ \begin{array}{ll} 1&{} \text {Master} \\ 0&{} \text {Others} \end{array}\right.$$
	$$x_{22}=\left\{ \begin{array}{ll} 1&{}\text {Doctor} \\ 0&{} \text {Others} \end{array}\right.$$
	Group undergraduate students set as dummy variable basic type (Anchoring group)
Place of birth	$$x_{3}=\left\{ \begin{array}{ll} 1&{} \text {City district} \\ 0&{} \text {Not in the city district} \end{array}\right.$$
Only child	$$x_{4}=\left\{ \begin{array}{ll} 1&{} \text {Yes} \\ 0&{} \text {No} \end{array}\right.$$
Level of Feminist Identity	$x_5$

### The influence of the level of feminist identity on childbearing desires

The results in the first column of Tables 4 and 5 show that the higher the level of feminist identity, the lower intensity of desire to have children and the lower child-number desires, which means that feminist identity has a significant negative effect on both the intensity of desire to have children and the child-number desires, that is, the higher the level of feminist identity the lower childbearing desires of the group.

**Table 4:** Multinomial logistic regression results

Intensity of desire to have children	Regression coefficient	Standard Error	Wald	df	P
Constant	0.023	0.361	0.004	36	0.950
Age_20-23	-0.59	0.346	2.928	36	0.087*
Age_24-26	0.653	0.225	8.442	36	0.004***
Age_27-29	0.627	0.685	0.839	36	0.360
Age_over 30 years old	0.215	0.461	0.218	36	0.640
Education_Master	0.713	0.292	5.967	36	0.015**
Education_Doctor	0.554	0.438	1.603	36	0.205
Place of birth	0.438	0.191	5.251	36	0.022**
Only child	-0.27	0.191	1.992	36	0.158
Level of Feminist Identity	-0.32	0.103	9.959	36	0.002***

Note: *p < 0.10, **p < 0.05, ***p < 0.01

**Table 5:** Ordinal logistic regression results

Child-number desires	Regression coefficient	Standard Error	z	P
Age_20-23	0.027	0.137	0.194	0.846
Age_24-26	-0.341	0.218	-1.561	0.118
Age_27-29	-0.094	0.483	-0.195	0.845
Age_over 30 years old	0.335	0.337	0.995	0.320
Education_Master	0.471	0.216	2.18	0.029**
Education_Doctor	0.608	0.303	2.01	0.044**
Place of birth	0.067	0.123	0.544	0.587
Only child	-0.549	0.125	-4.391	0.000***
Level of Feminist Identity	-0.7	0.07	-9.996	0.000***

Note: *p < 0.10, **p < 0.05, ***p < 0.01

### The influence of psychosocial factors on fertility intentions

After data pre-processing, reliability testing and normality testing, a structural equation model was developed. The latent variables are not directly observable, so this study, from a theoretical perspective, combined with the questions set out in the questionnaire, selected perceptions of fertility supports, perceptions of fertility hindrances and feminist identity as primary latent variables, and economic supports, physical supports, service provisions, timing supports and employment protections as secondary latent variables, and realised the consideration of latent variables through various types of observable variables, and the corresponding measurable settings and corresponding questions are shown in Table 6.

**Table 6:** Latent variable setting

Latent variables	Observed Variables	Description of the observed variables
Perceptions of fertility supports	Economic supports	I think stronger maternity insurance/more housing benefits/extended compulsory education will be more likely to increase my childbearing desires
	Physical supports	I think the government’s provision of more maternal facilities/the government’s provision of more children’s facilities/Employer’s provision of pregnant women and breastfeeding lounges will be more likely to increase my childbearing desires
	Timing supports	I think that extending the length of maternity leave, prenatal leave, maternity leave and breastfeeding leave / introducing parental leave for fathers or extending paternity leave / allowing female workers to telecommute during pregnancy will be more likely to increase my childbearing desires
	Service provisions	I think more government funding for maternity care and costs / subsidies from my employer when employees purchase services / psychological support from my employer / intergenerational parenting support and moral encouragement from our parents will be more likely to increase my childbearing desires
	Employment protections	I think that employers don’t restrict women’s wages, salaries, career development and marital status on the basis of gender/protect women’s work and employment after childbirth/protect women’s safety and health when working and labouring during special periods such as pregnancy and childbirth/reduce the workload of female workers during special periods will be more likely to increase my childbearing desires
Perceptions of fertility hindrances	Economic hindrances	I think the financial pressure of raising children will reduce my childbearing desires
	Physical hindrances	I think that the low number of maternal facilities and children’s facilities in society will reduce my childbearing desires
	Timing hindrances	I think the lack of time and energy to raise children will reduce my childbearing desires
	Service hindrances	I think the fear of not having better family care and increased family conflict will reduce my childbearing desires
	Employment hindrances	I think the potential loss of job opportunities and education as a result of pregnancy, nursing and childcare will reduce my childbearing desires
	Other hindrances	I think that children constrain personal freedom and will reduce my childbearing desires
		I think children interfere with the couple’s life and will reduce my childbearing desires
		I think the impact on marriage and quality of life will reduce my childbearing desires
		I think the pain or risk of childbirth will reduce my childbearing desires
Childbearing desires	Intensity of desire to have children	Whether you want to have children
	Child-number desires	How many children do you think is the ideal number for an average family, regardless of fertility policies and other conditions
Childbearing intentions	Child-number intentions	How many children do you plan to have
	Intended timing of birth	If you are planning to have a baby, when will you choose to have it

**Fig. 4:**
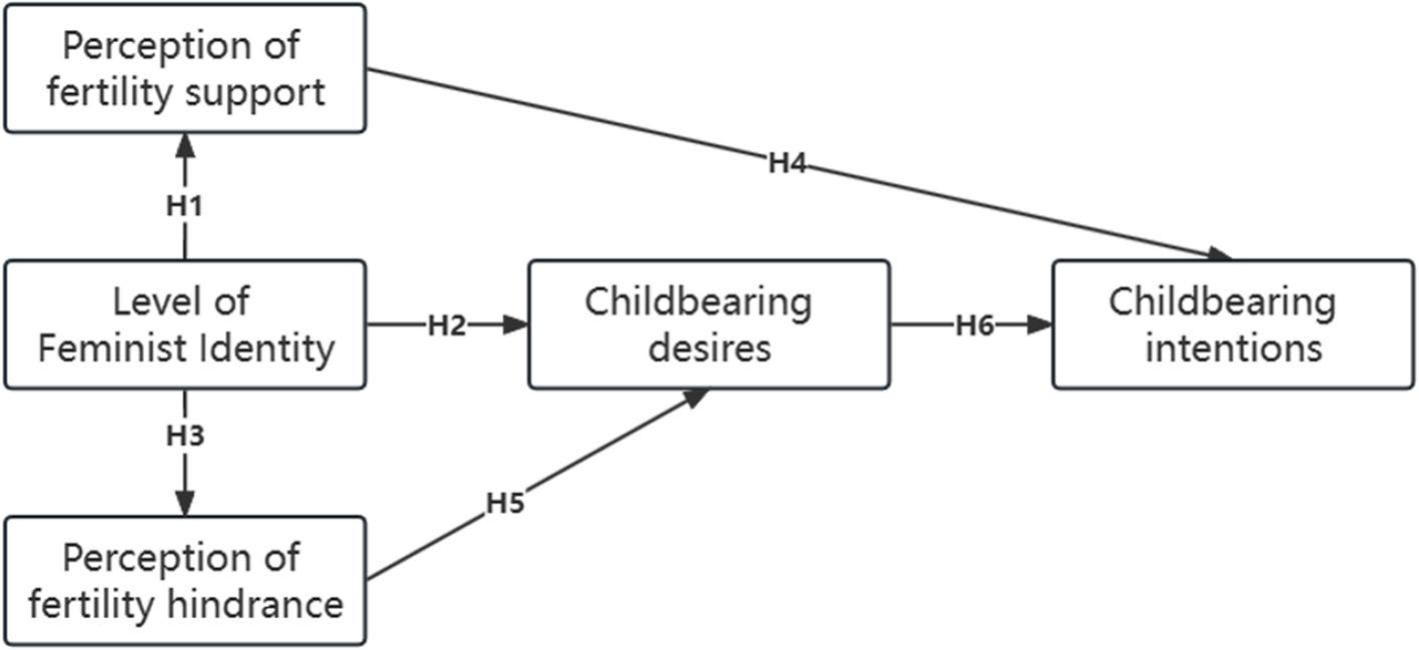
Conceptual diagram of the structural equation modeling

Based on the TDIB model, the hypothesis was formulated by combining life experience and literature survey, a conceptual diagram of the model (Fig. 4) with a table of relevant hypotheses (Table 7) was constructed and the pathway hypothesis was verified using AMOS software.

**Table 7:** Structural equation modeling related assumptions

	Model Assumptions
H1	The level of feminist identity positively influences perceptions of fertility supports
H2	The level of feminist identity positively influences childbearing desires
H3	The level of feminist identity positively influences perceptions of fertility hindrances
H4	The perceptions of fertility supports positively influences childbearing intentions
H5	The perceptions of fertility hindrances negatively influences childbearing desires
H6	Childbearing desires support positively influences childbearing intentions

**Fig. 5:**
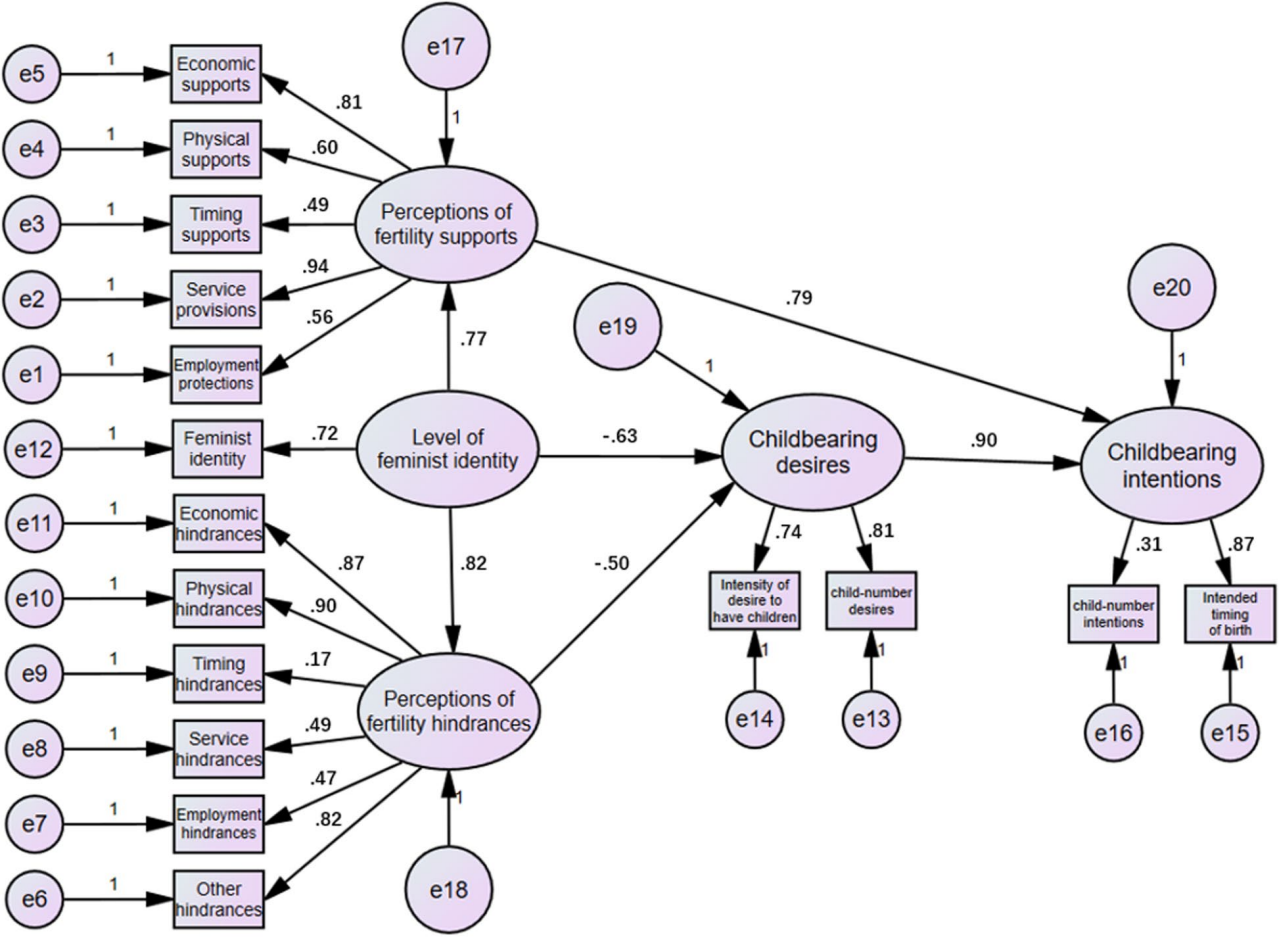
Structural equation modeling results graph

According to the above assumptions, we used AMOS 24.0 software to draw the causal path diagram of the model according to the notation rules of the structural equation model path diagram, specifying that one of the coefficients in the measurement index corresponding to each latent variable in the model is 1, which is equivalent to specifying that the unit of measure of the latent variable is the same as the unit of the corresponding measurement index; specifying that the measurable variables of the exogenous latent variable, endogenous latent variable The coefficient of measurement error is 1. The causal path is set up and the final results are calculated by the software as shown in Fig. 5, and the hypothesis tests are shown in Table 8.

**Table 8:** Structural equation modeling hypothesis testing results

Assumptions	Path relationships between latent variables			Estimate	P	Results
H1	Perceptions of fertility supports	<—	Level of Feminist Identity	0.77	0.028	True
H2	Childbearing desires	<—	Level of Feminist Identity	-0.63	0.009	True
H3	Perceptions of fertility hindrances	<—	Level of Feminist Identity	0.82	0.017	True
H4	Childbearing intentions	<—	Perceptions of fertility supports	0.79	0.000	True
H5	Childbearing desires	<—	Perceptions of fertility hindrances	-0.50	0.034	True
H6	Childbearing intentions	<—	Childbearing desires	0.90	0.000	True

The hypotheses H1, H2, H3, H4, H5 and H6 were tested and the results of the analysis were as follows:

The direct effect of the level of feminist identity on the perceptions of fertility supports and fertility hindrances among female university students was 0.77 and 0.82 respectively, indicating that the level of feminist identity had a positive effect on the perceptions of fertility supports and fertility hindrances among female university students. The higher the level of feminist identity, the stronger the perception of fertility supports and fertility hindrances.

Psychosocial factors had a greater degree of influence on fertility intentions. The direct effects of the level of feminist identity and the perception of fertility hindrances on childbearing desires are -0.63 and -0.50 respectively, and the direct effect of the perception of fertility supports on childbearing intentions is 0.79, showing that perceptions such as equal rights brought about by feminism and the perception of fertility hindrances such as economy, time, employment and services show a reduced utility on fertility intentions. The low fertility intentions of female university students are not only due to personal factors, but external barriers also have a significant negative impact on their fertility intentions, while providing financial, physical and service supports can effectively increase their fertility intentions.

The direct effect of childbearing desires on childbearing intentions was 0.90, the largest path coefficient. This indicates that the childbearing intentions of female university students are strongly and positively influenced by their own childbearing desires, and that the development from the germ of desires to intentions in their minds is in line with public perceptions of practice, and highlights the importance of raising people’s childbearing desires to the emergence of childbearing intentions and even fertility behaviours.

## Discussion

To our knowledge, this is the first study to examine the influence of feminist identity on fertility intentions in a large representative sample. Our findings show that the level of feminist identity has a significant negative effect on both the child-number desires and intensity of desire to have children. A growing amount of research has shown that motherhood penalties can cause mothers to experience disadvantages in terms of pay, benefits, and development opportunities due to childbearing [42–44], a situation that is more common in China [45–47], and that traditionalist fertility motivations that emphasise family interests and relationships have given way to individualist fertility motivations that emphasise personal emotional satisfaction, and which reinforces the notions of “having children for oneself” and “having fewer children later in life”, which are more likely to hinder the steady increase in fertility [48, 49]. A similar logic applies to the level of feminist identity: women with higher levels of feminist identity tend to delay marriage and childbearing while pursuing their careers, and the increasing opportunity costs discourage their fertility intentions [50, 51]. Therefore, our study further calls on the government to improve the status of women, strengthen the protection and support of women’s rights and interests, supervise employers not to restrict women’s wages, salaries, career development, marriage and childbearing on the basis of gender, reduce the workload of female workers during menstruation, pregnancy, childbirth, breastfeeding and other special periods, as well as safeguard women’s safety and health when they are working and labouring in these special periods, and at the same time safeguard women’s work and employment after giving birth, so that women can better balance childbirth and career development. At the same time, it protects women’s work and employment after childbirth, so that women can strike a better balance between childbirth and career development, and effectively safeguard women’s rights and the safety of their lives and health. Additionally, employers may create flexible work hours and improve employee benefits to lessen the financial strain and work pressure on staff members and make it simpler for them to combine work and family obligations, which will increase their willingness to become parents.

Our findings provide further evidence that psychosocial factors influence fertility intentions to a greater extent, with the level of feminist identification, the perception of fertility hindrances and the perception of fertility supports all having a significant impact on fertility intentions. Consistent with previous findings, the marginal role of fertility policy in guiding fertility behaviour has significantly diminished in China [52, 31, 53–55], and the fertility supports that female university students care most about have shifted from financial supports to employment protections, therefore, the government can formulate policies to alleviate the conflict between women’s childbirth and their careers by providing more policies, funding and training opportunities as a means to increase the income of the working population, thus reducing the concerns and worries of a family that is unable to afford the act of childbearing in terms of financial strength. The government can also reduce the work pressure and workload of commuters by promoting flexible working and supporting paid parental leave, thus making it easier for them to balance their work and family life and have more time and energy to take care of their families and children [56], meanwhile, it is necessary to provide a full range of social security to reduce the burden of childbearing and childcare on families by covering more of the costs of basic healthcare services and fees and providing more childcare services, making it easier for parents to balance work and family life [57].Employers should provide better employee benefits, implement flexi-work and in-home work to reduce the economic burden and work pressure of employees [58], as well as strengthen the health management of employees and offer professional psychological counselling services, so as to help them reduce the psychological pressure of the process of childbearing, and to maintain good physical and mental health, thus enhancing the confidence and determination of childbearing.

### Limitations

Three limitations of this study are worth mentioning. Firstly, the perceptions of fertility hindrances and fertility supports of the female university students in our study were based only on previous non-independent life experiences, which may have shifted significantly after entering society.In the future, the same research subjects can be followed up to reduce the resulting error. Secondly, the sample we used had not yet given birth to a child, and those female university students who were pregnant or had already given birth to a child were excluded from the study, which may have led to a degree of sample selection bias.This can be tested later by expanding the research subjects to include female university students who are pregnant and have given birth to children in the study. Thirdly, in generalising the findings, the sample from the three universities in Nanjing may not reflect the fertility intentions of female university students in other parts of China. In the future, more regions can be included for large sample comparison and tracking research.

## Conclusions

The psychosocial factors of fertility intentions and their pathways of influence among Chinese female university students were explored through data collected in Nanjing, China, from February to March 2023. Our findings show that the level of feminist identity has a significant negative effect on both the child-number desires and the intensity of desire to have children; the higher the level of feminist identity, the lower the intensity of desire to have children and the lower child-number desires. Further research shows that psychosocial factors have a greater impact on fertility intentions, with feminist identity, the perception of fertility hindrances and the perception of fertility supports all having a significant impact on fertility intentions. The government and employers need to take action to build a fertility-friendly society.

## Data Availability

The datasets used and/or analysed during the current study are available from the corresponding author on reasonable request.
